# Crystal structure and Hirshfeld surface analysis of 4-benzyl-2*H*-benzo[*b*][1,4]oxazin-3(4*H*)-one

**DOI:** 10.1107/S2056989025007686

**Published:** 2025-09-05

**Authors:** Soukaina El Haddad, Olivier Blacque, Tuncer Hökelek, Ahmed Mazzah, Taha Mohamed Labd, Lhoussaine El Ghayati, Nada Kheira Sebbar

**Affiliations:** a Laboratory of Heterocyclic Organic Chemistry Medicines Science Research Center Pharmacochemistry Competence Center Mohammed V University in Rabat Faculté des Sciences Av Ibn Battouta BP 1014 Rabat Morocco; b University of Zurich, Department of Chemistry, Winterthurerstrasse 190, CH-8057 Zurich, Switzerland; c Department of Physics, Hacettepe University, 06800 Beytepe, Ankara, Türkiye; d Science and Technology of Lille USR 3290, Villeneuve d’ascq cedex, France; e Laboratory of Organic and Physical Chemistry, Applied Bioorganic Chemistry Team, Faculty of Sciences, Ibnou Zohr University, Agadir, Morocco; Vienna University of Technology, Austria

**Keywords:** crystal structure, heterocycles, 1,4-benzoxazin-3-ones, C—H⋯π inter­action.

## Abstract

The mol­ecule of the title compound, C_15_H_13_NO_2_, comprises a non-planar oxazine ring (twisted–boat conformation) fused to a benzene ring, and a benzyl moiety. In the crystal, C—H⋯O hydrogen bonds link the mol­ecules into [010] supra­molecular chains, enclosing *R*_2_^2^(9) ring motifs. C—H⋯π inter­actions and very weak π–π stacking between the benzene rings of adjacent mol­ecules help to consolidate the packing. A Hirshfeld surface analysis revealed that the most important contributions to the crystal packing are from H⋯H (48.8%), H⋯ C/C⋯H (29.3%) and H⋯O/O⋯H (18.9%) inter­actions.

## Abstract 2

The title mol­ecule contains a non-planar oxazine and two benzene rings, with the oxazine ring in a twisted-boat conformation.

## 1. Chemical context

**Figure 1:**
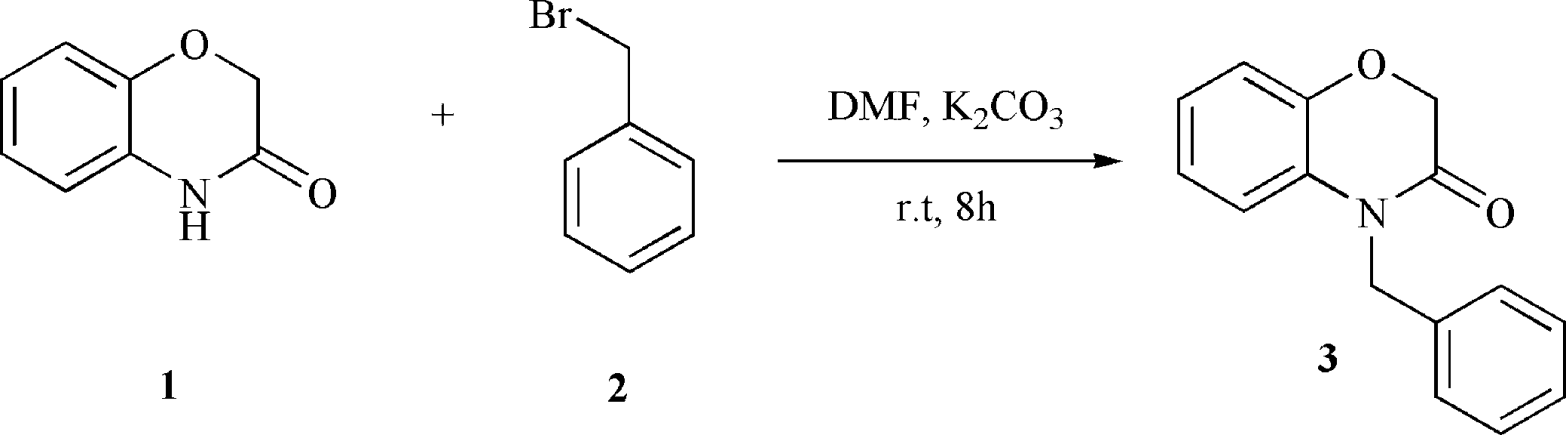
Reaction scheme for obtaining the title compound, **3**.

1,4-Benzoxazine and its derivatives are heterocycles, resulting from the fusion of a benzene ring with an oxazine ring, containing O and N heteroatoms in positions 1 and 4, respectively. These structural elements give these compounds high chemical reactivity and are of particular inter­est for the development of bioactive mol­ecules. Among them, 1,4-benzoxazin-3-ones constitute a subclass with a wide range of pharmacological properties, including anti­tumor, anti­bacterial, anti­fungal, anti­cancer, anti­viral and anti­depressant activities (Hlimi *et al.*, 2018 ▸; Oksuzoglu *et al.*, 2023 ▸; Tang *et al.*, 2023 ▸; Fringuelli *et al.*, 2002 ▸; Benarjee & Saritha, 2022 ▸; Rao *et al.*, 2022 ▸; Zhou *et al.*, 2006 ▸). As part of our research on benzoxazines (Sebbar *et al.*, 2025 ▸), we developed a selective alkyl­ation strategy aimed at introducing a benzyl group, **2**, onto the 1,4-benzoxazin-3-one nucleus, **1**. This transformation was carried out in a polar aprotic solvent (di­methyl­formamide, DMF), in the presence of potassium carbonate (K_2_CO_3_) as a base, allowing for an efficient reaction with a halogenated derivative (Fig. 1 ▸). This methodology enables the targeted production of new functionalized derivatives for future structural and biological evaluations and led to the synthesis of 4-benzyl-2*H*-benzo[*b*][1,4]oxazin-3(4*H*)-one (C_15_H_13_NO_2_), **3**, in 90% yield. We report here on the mol­ecular and crystal structures of this compound and also present the results of its Hirshfeld surface analysis.
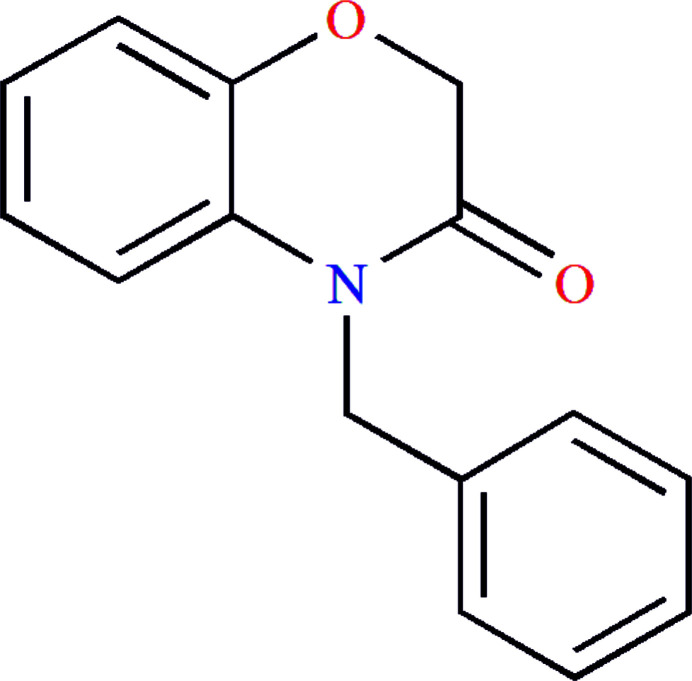


## 2. Structural commentary

**Figure 2:**
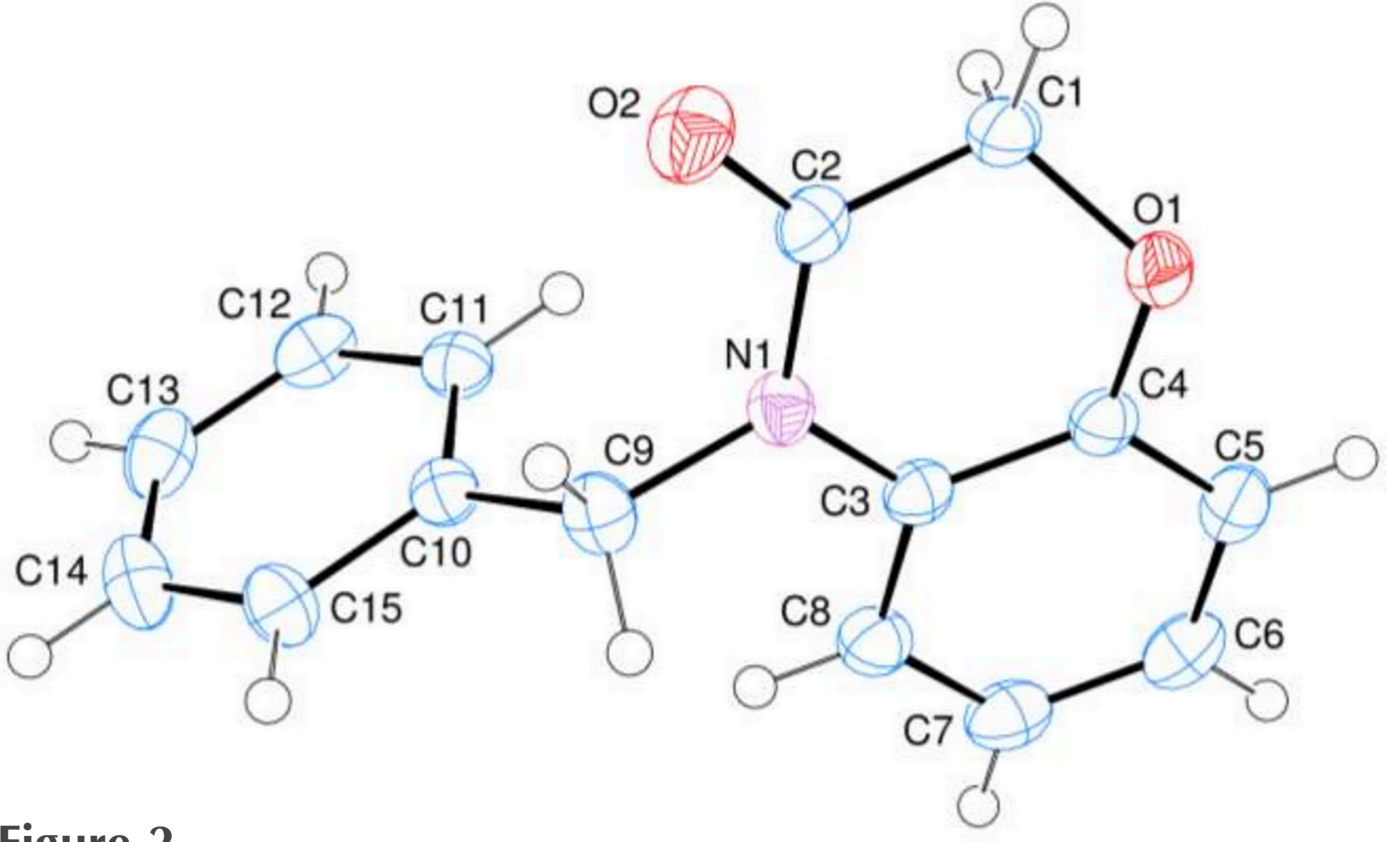
The mol­ecular structure of **3** showing displacement ellipsoids at the 50% probability level.

**Figure 3:**
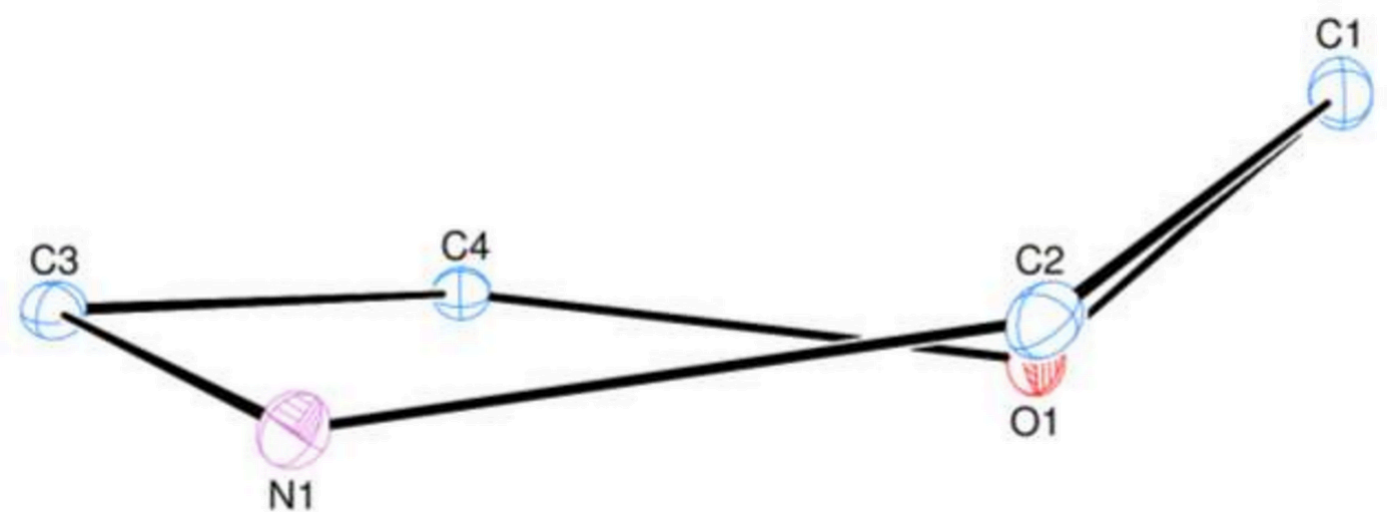
Conformation of the oxazine ring atoms.

Compound **3** contains a non-planar oxazine ring fused to a benzene ring, and a benzyl moiety (Fig. 2 ▸). The oxazine ring *A* (O1/N1/C1–C4) has a twisted-boat conformation (Fig. 3 ▸) with puckering parameters (Cremer & Pople, 1975 ▸) *Q*_T_ = 0.4286 (10) Å, θ = 112.77 (13)° and φ = 213.01 (15)°. The benzene rings *B* (C3–C8) and *C* (C10–C15) are oriented at a dihedral angle of 87.27 (3)°, and atom C9 is displaced by −0.1060 (11) Å from the mean plane of ring *C*. Bond lengths and angles appear to be in normal ranges.

## 3. Supra­molecular features

**Figure 4:**
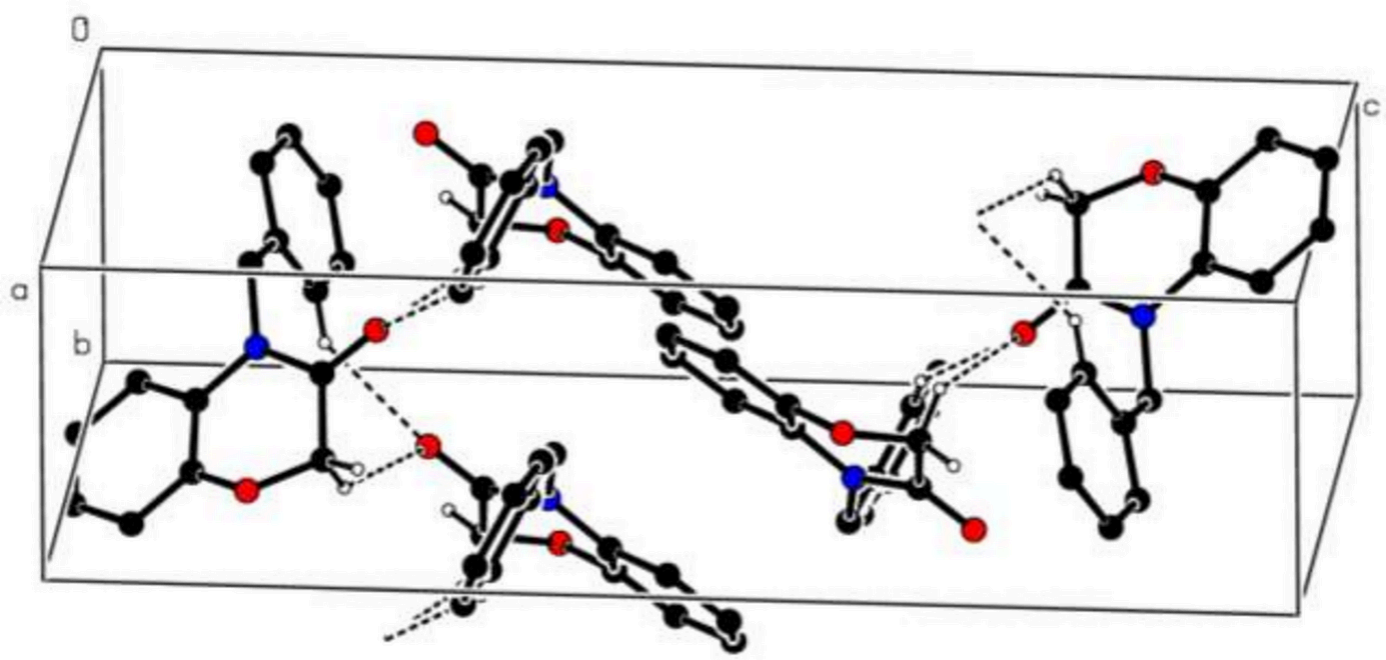
Partial packing diagram of **3** with inter­molecular C—H⋯O hydrogen bonds shown as dashed lines; only those hydrogen atoms involved in these inter­actions are shown.

In the crystal of **3**, C1—H1*A*⋯O2^i^ and C11—H11⋯O2^i^ hydrogen bonds (Table 1 ▸) link adjacent mol­ecules into [010] supra­molecular chains, enclosing 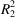
(9) ring motifs (Etter *et al.*, 1990 ▸), Fig. 4 ▸. An additional C—H⋯π(ring) inter­action (Table 1 ▸) and a very weak π–π stacking inter­action between the *B* rings of adjacent mol­ecules with a centroid-to-centroid distance of 4.0255 (6) Å, a dihedral angle *α* of 0.02 (5)° and a slippage of 2.291 Å help to consolidate the packing within the crystal.

## 4. Hirshfeld surface analysis

**Figure 5:**
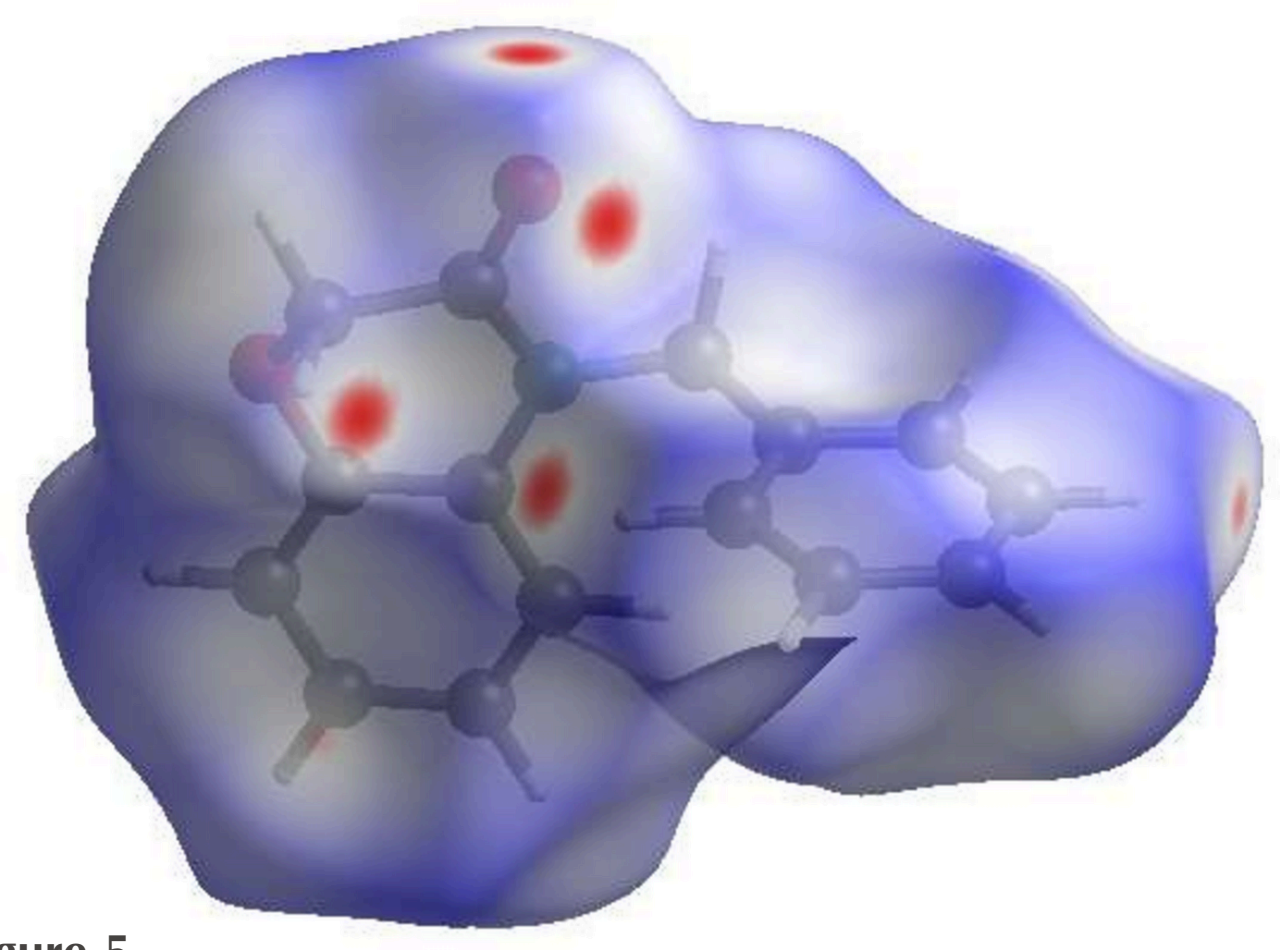
View of the three-dimensional HS of **3** plotted over *d*_norm_.

**Figure 6:**
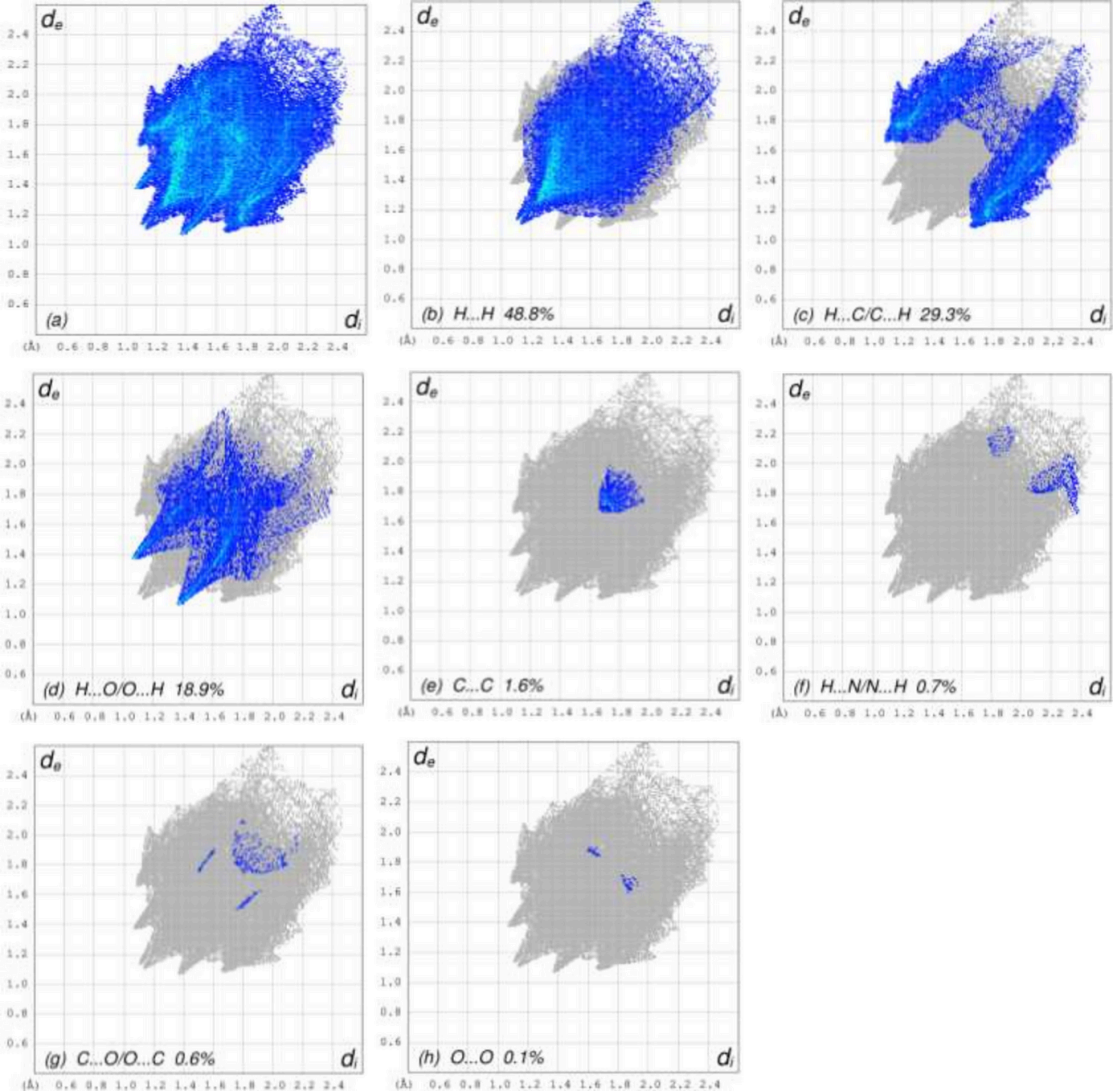
The two-dimensional fingerprint plots of **3**, showing (*a*) all inter­actions, and delineated into (*b*) H⋯H, (*c*) H⋯C/C⋯H, (*d*) H⋯O/O⋯H, (*e*) C⋯C, (*f*) H⋯N/N⋯H, (*g*) C⋯O/O⋯C and (*h*) O⋯O inter­actions. The *d*_i_ and *d*_e_ values are the closest inter­nal and external distances (in Å) from given points on the Hirshfeld surface.

To qu­antify and visualize the inter­molecular inter­actions in the crystal of **3**, a Hirshfeld surface (HS) analysis was carried out with *CrystalExplorer* (Spackman *et al.*, 2021 ▸). Fig. 5 ▸ shows the contact distances on the HS where the bright-red spots correspond to the respective donors and/or acceptor sites noted above. According to the two-dimensional fingerprint plots (McKinnon *et al.*, 2007 ▸), H⋯H, H⋯C/C⋯H and H⋯O/O⋯H contacts make the most significant contributions to the HS, at 48.8%, 29.3% and 18.9%, respectively (Fig. 6 ▸).

## 5. Database survey

**Figure 7:**
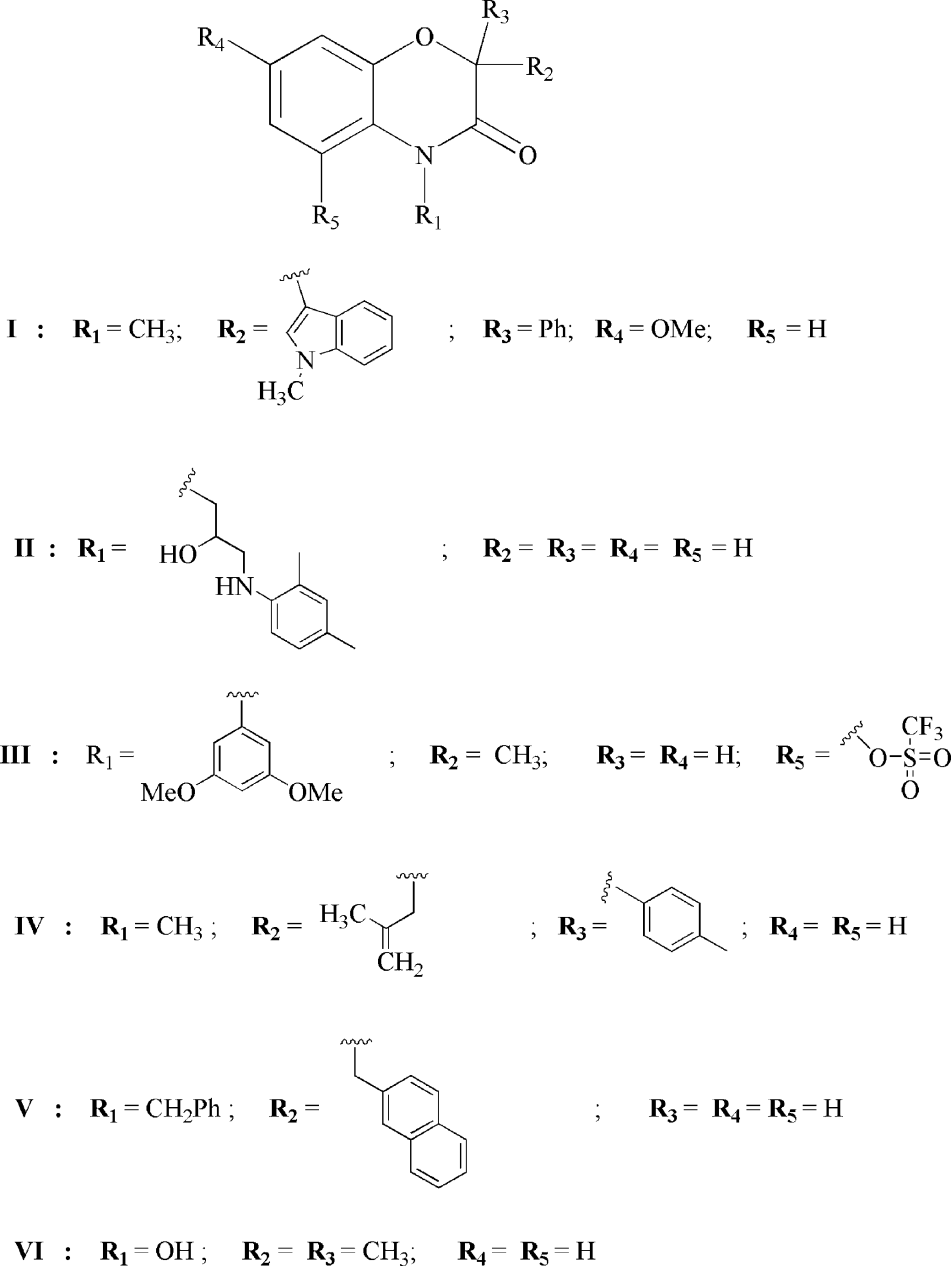
Results of the database search with the closest structures identified.

A search of the Cambridge Structural Database (CSD, updated July 2025; Groom *et al.*, 2016 ▸) for compounds with the benzo[b][1,4]oxazin-3(4H)-one moiety substituted in the 2-, 4-, 5-, or 7-positions revealed numerous entries. The compounds most closely related to **3** are schematically displayed in Fig. 7 ▸ and include: Structures **I** (CSD refcode DAMYOJ; Shaikh *et al.*, 2021 ▸); **II** (BUHROO, with ***R***_1_ = 4-(3-(2,4-di­methyl­phenyl­amino)­propan-2-ol­yl), ***R*****_2_** to ***R*****_5_** = H; Rao *et al.*, 2020 ▸); **III** (DUFKEX; Yang *et al.*, 2019 ▸); **IV** (HAHCEC, with ***R***_1_ = CH_3_, ***R*****_2_** = 2-(2-methyl­prop-1-en­yl), ***R***_3_ = 2-(4- methyl­benz­yl), ***R*****_4_** = ***R*****_5_** = H; Mohanta *et al.*, 2023 ▸); **V** (INICIU; Nie *et al.*, 2021 ▸) and **VI** (KOQSES; Winter *et al.*, 2024 ▸). In comparison, the title compound **3** is N-benzyl­ated and unsubstituted on the ring (***R*****_2_ –*****R*****_5_** = H) and thus is distinguished by a moderate bulky group at the nitro­gen atom and greater rotational freedom of the –CH_2_–Ph arm, conditions favorable to (weak) π–π stacking and C—H⋯O contacts. Conversely, **II** and **VI** carry polar functions (*e.g.*, alcohol/amine or hydroxyl groups) to establish O—H⋯O / N—H⋯O networks influencing the local conformation. **III** introduces a strongly electron-withdrawing group (tri­fluoro­methyl sulfon­yl), which strengthens the C—H⋯O contacts and modifies the electron density of oxazinone. **I**, **IV**, and **V** present bulky/aromatic substituents (N-alkyls, aryls), inducing more pronounced shifts (slippage) in the π stacks and higher N—C torsion angles, which result in distinct packing modes. The conformation of the oxazine ring in these structures is similar to that observed in the title compound.

## 6. Synthesis and crystallization

A dry mixture was prepared by combining 100 mg (0.6 mmol) of 2*H*-benzo[*b*][1,4]oxazin-3(4*H*)-one (previously dried under vacuum in a desiccator) with 92.1 mg (0.66 mmol) of potassium carbonate (K_2_CO_3_) in 15 ml of anhydrous DMF. To this mixture, 0.66 mmol of benzyl bromide were added. The reaction mixture was stirred at room temperature, and the reaction progress was monitored using thin-layer chromatography (TLC). Once the reaction was complete, the mixture was filtered to remove inorganic salts, and the solvent was evaporated under reduced pressure. The crude product was then purified using silica gel column chromatography, employing a mixture of hexane and ethyl acetate as the eluent. The target compound **3** was obtained as colorless crystals with an overall yield of 90%.

## 7. Refinement

Crystal data, data collection and structure refinement details are summarized in Table 2 ▸. C-bound hydrogen atoms were calculated geometrically at CH = 0.95 Å and CH_2_ = 0.99 Å and refined using a riding model with *U*_iso_(H) = 1.2*U*_eq_(C).

## Supplementary Material

Crystal structure: contains datablock(s) I. DOI: 10.1107/S2056989025007686/wm5767sup1.cif

Structure factors: contains datablock(s) I. DOI: 10.1107/S2056989025007686/wm5767Isup2.hkl

Supporting information file. DOI: 10.1107/S2056989025007686/wm5767Isup3.cdx

Supporting information file. DOI: 10.1107/S2056989025007686/wm5767Isup4.cml

CCDC reference: 2483523

Additional supporting information:  crystallographic information; 3D view; checkCIF report

## Figures and Tables

**Table 1:** Hydrogen-bond geometry (Å, °) *Cg*3 is the centroid of the C10–C15 ring.

*D*—H⋯*A*	*D*—H	H⋯*A*	*D*⋯*A*	*D*—H⋯*A*
C1—H1*A*⋯O2^i^	0.99	2.50	3.2592 (12)	133
C11—H11⋯O2^i^	0.95	2.56	3.3765 (13)	146
C1—H1*B*⋯*Cg*3^i^	0.99	2.78	3.5952 (10)	143

Symmetry code: (i) 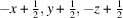.

**Table 2:** Experimental details

Crystal data	
Chemical formula	C_15_H_13_NO_2_
*M*_r_	239.26
Crystal system, space group	Monoclinic, *P*2_1_/*n*
Temperature (K)	160
*a*, *b*, *c* (Å)	9.49281 (8), 5.79368 (5), 21.45658 (18)
β (°)	91.3621 (8)
*V* (Å^3^)	1179.74 (2)
*Z*	4
Radiation type	Cu *K*α
μ (mm^−1^)	0.73
Crystal size (mm)	0.30 × 0.13 × 0.11

Data collection	
Diffractometer	XtaLAB Synergy, Dualflex, HyPix
Absorption correction	Analytical (*CrysAlis PRO*; Rigaku OD, 2024 ▸)
*T*_min_, *T*_max_	0.859, 0.929
No. of measured, independent and observed [*I* > 2σ(*I*)] reflections	15357, 2477, 2412
*R*_int_	0.013
(sin θ/λ)_max_ (Å^−1^)	0.633

Refinement	
*R*[*F*^2^ > 2σ(*F*^2^)], *wR*(*F*^2^), *S*	0.034, 0.089, 1.04
No. of reflections	2477
No. of parameters	163
H-atom treatment	H-atom parameters constrained
Δρ_max_, Δρ_min_ (e Å^−3^)	0.17, −0.19

Computer programs: *CrysAlis PRO* (Rigaku OD, 2024 ▸), *SHELXT* (Sheldrick, 2015*a* ▸), *SHELXL* (Sheldrick, 2015*b* ▸), *OLEX2* (Dolomanov *et al.*, 2009 ▸) and *publCIF* (Westrip, 2010 ▸).

## supplementary crystallographic information

### 4-Benzyl-2H-benzo[b][1,4]oxazin-3(4H)-one Crystal data

**Table:**

C_15_H_13_NO_2_	*F*(000) = 504
*M**_r_* = 239.26	*D*_x_ = 1.347 Mg m^−^^3^
Monoclinic, *P*2_1_/*n*	Cu *K*α radiation, λ = 1.54184 Å
*a* = 9.49281 (8) Å	Cell parameters from 12385 reflections
*b* = 5.79368 (5) Å	θ = 4.1–79.3°
*c* = 21.45658 (18) Å	µ = 0.73 mm^−^^1^
β = 91.3621 (8)°	*T* = 160 K
*V* = 1179.74 (2) Å^3^	Block, colourless
*Z* = 4	0.30 × 0.13 × 0.11 mm

### 4-Benzyl-2H-benzo[b][1,4]oxazin-3(4H)-one Data collection

**Table:**

XtaLAB Synergy, Dualflex, HyPix diffractometer	2477 independent reflections
Radiation source: micro-focus sealed X-ray tube, PhotonJet (Cu) X-ray Source	2412 reflections with *I* > 2σ(*I*)
Mirror monochromator	*R*_int_ = 0.013
Detector resolution: 10.0000 pixels mm^-1^	θ_max_ = 77.3°, θ_min_ = 4.1°
ω scans	*h* = −11→11
Absorption correction: analytical (CrysAlisPro; Rigaku OD, 2024)	*k* = −6→7
*T*_min_ = 0.859, *T*_max_ = 0.929	*l* = −27→25
15357 measured reflections	

### 4-Benzyl-2H-benzo[b][1,4]oxazin-3(4H)-one Refinement

**Table:**

Refinement on *F*^2^	Primary atom site location: dual
Least-squares matrix: full	Hydrogen site location: inferred from neighbouring sites
*R*[*F*^2^ > 2σ(*F*^2^)] = 0.034	H-atom parameters constrained
*wR*(*F*^2^) = 0.089	*w* = 1/[σ^2^(*F*_o_^2^) + (0.045*P*)^2^ + 0.3423*P*] where *P* = (*F*_o_^2^ + 2*F*_c_^2^)/3
*S* = 1.04	(Δ/σ)_max_ < 0.001
2477 reflections	Δρ_max_ = 0.17 e Å^−^^3^
163 parameters	Δρ_min_ = −0.19 e Å^−^^3^
0 restraints	

### 4-Benzyl-2H-benzo[b][1,4]oxazin-3(4H)-one Special details

**Table:**

Geometry. All esds (except the esd in the dihedral angle between two l.s. planes) are estimated using the full covariance matrix. The cell esds are taken into account individually in the estimation of esds in distances, angles and torsion angles; correlations between esds in cell parameters are only used when they are defined by crystal symmetry. An approximate (isotropic) treatment of cell esds is used for estimating esds involving l.s. planes.

### 4-Benzyl-2H-benzo[b][1,4]oxazin-3(4H)-one Fractional atomic coordinates and isotropic or equivalent isotropic displacement parameters (Å^2^)

**Table:**

	*x*	*y*	*z*	*U*_iso_*/*U*_eq_	
O1	−0.00508 (7)	0.54481 (13)	0.36181 (3)	0.02998 (18)	
O2	0.15569 (8)	0.13347 (15)	0.26572 (4)	0.0379 (2)	
N1	0.22040 (8)	0.24375 (14)	0.36413 (4)	0.02528 (19)	
C1	0.05704 (10)	0.48342 (19)	0.30398 (4)	0.0284 (2)	
H1A	0.115402	0.613732	0.289607	0.034*	
H1B	−0.018786	0.457366	0.272281	0.034*	
C2	0.14752 (10)	0.26962 (18)	0.30884 (5)	0.0268 (2)	
C3	0.20721 (10)	0.40933 (17)	0.41222 (4)	0.0242 (2)	
C4	0.09204 (10)	0.55889 (17)	0.41010 (4)	0.0251 (2)	
C5	0.06932 (11)	0.71387 (19)	0.45779 (5)	0.0319 (2)	
H5	−0.010120	0.813576	0.455994	0.038*	
C6	0.16316 (12)	0.7230 (2)	0.50828 (5)	0.0357 (3)	
H6	0.147692	0.828356	0.541354	0.043*	
C7	0.27928 (12)	0.5787 (2)	0.51046 (5)	0.0356 (3)	
H7	0.344034	0.586946	0.544835	0.043*	
C8	0.30189 (11)	0.42197 (19)	0.46287 (5)	0.0308 (2)	
H8	0.381803	0.323285	0.464743	0.037*	
C9	0.30952 (10)	0.04000 (17)	0.37316 (5)	0.0289 (2)	
H9A	0.272671	−0.084600	0.345729	0.035*	
H9B	0.300835	−0.012997	0.416763	0.035*	
C10	0.46435 (10)	0.07306 (16)	0.36029 (4)	0.0239 (2)	
C11	0.51780 (10)	0.26787 (17)	0.33164 (4)	0.0264 (2)	
H11	0.457398	0.393858	0.321551	0.032*	
C12	0.66043 (11)	0.2784 (2)	0.31765 (5)	0.0323 (2)	
H12	0.697103	0.412364	0.298315	0.039*	
C13	0.74884 (11)	0.0947 (2)	0.33182 (5)	0.0359 (3)	
H13	0.845560	0.101544	0.321565	0.043*	
C14	0.69600 (12)	−0.0988 (2)	0.36092 (6)	0.0370 (3)	
H14	0.756507	−0.224714	0.370939	0.044*	
C15	0.55472 (11)	−0.10865 (18)	0.37543 (5)	0.0317 (2)	
H15	0.519116	−0.240940	0.395942	0.038*	

### 4-Benzyl-2H-benzo[b][1,4]oxazin-3(4H)-one Atomic displacement parameters (Å^2^)

**Table:**

	*U*^11^	*U*^22^	*U*^33^	*U*^12^	*U*^13^	*U*^23^
O1	0.0217 (3)	0.0411 (4)	0.0271 (4)	0.0047 (3)	0.0009 (3)	−0.0012 (3)
O2	0.0347 (4)	0.0444 (5)	0.0346 (4)	−0.0014 (3)	−0.0012 (3)	−0.0142 (3)
N1	0.0218 (4)	0.0267 (4)	0.0274 (4)	0.0008 (3)	0.0003 (3)	−0.0016 (3)
C1	0.0239 (5)	0.0378 (6)	0.0236 (5)	0.0006 (4)	−0.0001 (3)	0.0005 (4)
C2	0.0206 (4)	0.0331 (5)	0.0269 (5)	−0.0043 (4)	0.0019 (3)	−0.0028 (4)
C3	0.0231 (4)	0.0266 (5)	0.0232 (4)	−0.0032 (4)	0.0031 (3)	0.0015 (4)
C4	0.0226 (4)	0.0286 (5)	0.0242 (5)	−0.0026 (4)	0.0031 (3)	0.0025 (4)
C5	0.0322 (5)	0.0316 (5)	0.0324 (5)	0.0007 (4)	0.0081 (4)	−0.0013 (4)
C6	0.0433 (6)	0.0366 (6)	0.0276 (5)	−0.0062 (5)	0.0075 (4)	−0.0061 (4)
C7	0.0365 (5)	0.0453 (6)	0.0249 (5)	−0.0078 (5)	−0.0019 (4)	−0.0005 (4)
C8	0.0269 (5)	0.0377 (6)	0.0276 (5)	−0.0009 (4)	−0.0013 (4)	0.0020 (4)
C9	0.0266 (5)	0.0234 (5)	0.0366 (5)	−0.0011 (4)	0.0024 (4)	0.0018 (4)
C10	0.0247 (5)	0.0236 (4)	0.0235 (4)	−0.0007 (4)	−0.0009 (3)	−0.0025 (3)
C11	0.0287 (5)	0.0256 (5)	0.0248 (4)	−0.0015 (4)	−0.0014 (4)	−0.0005 (4)
C12	0.0330 (5)	0.0356 (6)	0.0283 (5)	−0.0093 (4)	0.0034 (4)	−0.0024 (4)
C13	0.0239 (5)	0.0450 (6)	0.0389 (6)	−0.0031 (4)	0.0028 (4)	−0.0126 (5)
C14	0.0290 (5)	0.0333 (6)	0.0483 (6)	0.0067 (4)	−0.0043 (4)	−0.0074 (5)
C15	0.0314 (5)	0.0244 (5)	0.0391 (6)	0.0012 (4)	−0.0011 (4)	0.0008 (4)

### 4-Benzyl-2H-benzo[b][1,4]oxazin-3(4H)-one Geometric parameters (Å, º)

**Table:**

O1—C1	1.4313 (12)	C7—C8	1.3869 (16)
O1—C4	1.3729 (12)	C8—H8	0.9500
O2—C2	1.2197 (13)	C9—H9A	0.9900
N1—C2	1.3671 (13)	C9—H9B	0.9900
N1—C3	1.4167 (12)	C9—C10	1.5140 (13)
N1—C9	1.4626 (12)	C10—C11	1.3871 (14)
C1—H1A	0.9900	C10—C15	1.3916 (14)
C1—H1B	0.9900	C11—H11	0.9500
C1—C2	1.5096 (14)	C11—C12	1.3951 (14)
C3—C4	1.3949 (14)	C12—H12	0.9500
C3—C8	1.3957 (14)	C12—C13	1.3847 (16)
C4—C5	1.3822 (14)	C13—H13	0.9500
C5—H5	0.9500	C13—C14	1.3831 (17)
C5—C6	1.3872 (16)	C14—H14	0.9500
C6—H6	0.9500	C14—C15	1.3852 (15)
C6—C7	1.3836 (17)	C15—H15	0.9500
C7—H7	0.9500		
			
C4—O1—C1	112.70 (7)	C3—C8—H8	120.0
C2—N1—C3	120.41 (8)	C7—C8—C3	119.97 (10)
C2—N1—C9	118.86 (8)	C7—C8—H8	120.0
C3—N1—C9	120.72 (8)	N1—C9—H9A	108.3
O1—C1—H1A	109.0	N1—C9—H9B	108.3
O1—C1—H1B	109.0	N1—C9—C10	115.77 (8)
O1—C1—C2	112.92 (8)	H9A—C9—H9B	107.4
H1A—C1—H1B	107.8	C10—C9—H9A	108.3
C2—C1—H1A	109.0	C10—C9—H9B	108.3
C2—C1—H1B	109.0	C11—C10—C9	123.41 (9)
O2—C2—N1	123.18 (10)	C11—C10—C15	119.28 (9)
O2—C2—C1	121.64 (9)	C15—C10—C9	117.22 (9)
N1—C2—C1	115.17 (8)	C10—C11—H11	120.1
C4—C3—N1	118.66 (9)	C10—C11—C12	119.87 (9)
C4—C3—C8	118.86 (9)	C12—C11—H11	120.1
C8—C3—N1	122.43 (9)	C11—C12—H12	119.8
O1—C4—C3	119.97 (9)	C13—C12—C11	120.36 (10)
O1—C4—C5	118.92 (9)	C13—C12—H12	119.8
C5—C4—C3	121.02 (9)	C12—C13—H13	120.1
C4—C5—H5	120.2	C14—C13—C12	119.84 (10)
C4—C5—C6	119.63 (10)	C14—C13—H13	120.1
C6—C5—H5	120.2	C13—C14—H14	120.1
C5—C6—H6	120.0	C13—C14—C15	119.89 (10)
C7—C6—C5	119.97 (10)	C15—C14—H14	120.1
C7—C6—H6	120.0	C10—C15—H15	119.6
C6—C7—H7	119.7	C14—C15—C10	120.74 (10)
C6—C7—C8	120.52 (10)	C14—C15—H15	119.6
C8—C7—H7	119.7		
			
O1—C1—C2—O2	145.86 (9)	C4—C3—C8—C7	1.08 (15)
O1—C1—C2—N1	−35.32 (12)	C4—C5—C6—C7	0.48 (16)
O1—C4—C5—C6	177.15 (9)	C5—C6—C7—C8	−0.89 (17)
N1—C3—C4—O1	−0.48 (13)	C6—C7—C8—C3	0.10 (16)
N1—C3—C4—C5	175.91 (9)	C8—C3—C4—O1	−177.89 (8)
N1—C3—C8—C7	−176.23 (9)	C8—C3—C4—C5	−1.50 (14)
N1—C9—C10—C11	−10.84 (14)	C9—N1—C2—O2	−1.88 (14)
N1—C9—C10—C15	172.60 (9)	C9—N1—C2—C1	179.31 (8)
C1—O1—C4—C3	−34.64 (12)	C9—N1—C3—C4	−160.52 (9)
C1—O1—C4—C5	148.89 (9)	C9—N1—C3—C8	16.79 (13)
C2—N1—C3—C4	18.13 (13)	C9—C10—C11—C12	−175.70 (9)
C2—N1—C3—C8	−164.55 (9)	C9—C10—C15—C14	175.23 (10)
C2—N1—C9—C10	96.66 (11)	C10—C11—C12—C13	0.47 (15)
C3—N1—C2—O2	179.44 (9)	C11—C10—C15—C14	−1.48 (15)
C3—N1—C2—C1	0.63 (13)	C11—C12—C13—C14	−1.08 (16)
C3—N1—C9—C10	−84.67 (11)	C12—C13—C14—C15	0.41 (16)
C3—C4—C5—C6	0.72 (15)	C13—C14—C15—C10	0.88 (17)
C4—O1—C1—C2	51.88 (11)	C15—C10—C11—C12	0.80 (14)

### 4-Benzyl-2H-benzo[b][1,4]oxazin-3(4H)-one Hydrogen-bond geometry (Å, º)

*Cg*3 is the centroid of the C10–C15 ring.

**Table:**

*D*—H···*A*	*D*—H	H···*A*	*D*···*A*	*D*—H···*A*
C1—H1*A*···O2^i^	0.99	2.50	3.2592 (12)	133
C11—H11···O2^i^	0.95	2.56	3.3765 (13)	146
C1—H1*B*···*Cg*3^i^	0.99	2.78	3.5952 (10)	143

Symmetry code: (i) −*x*+1/2, *y*+1/2, −*z*+1/2.
